# Triple Therapy in COPD in Real Life: Is It Better to Use Single or Multiple Inhalers?

**DOI:** 10.3390/jcm13206191

**Published:** 2024-10-17

**Authors:** Bruno Sposato, Alberto Ricci, Leonardo Gianluca Lacerenza, Elisa Petrucci, Alberto Cresti, Pasquale Baratta, Antonio Perrella, Andrea Serafini, Marco Scalese

**Affiliations:** 1 Pneumology Department, Local Health Unit ‘Sud-Est’, ‘Misericordia’ Hospital, 58100 Grosseto, Italy; antonio.perrella@uslsudest.toscana.it; 2 Division of Pneumology, Department of Clinical and Molecular Medicine, Sapienza University of Rome, AOU Sant‘Andrea, 00189 Rome, Italy; alberto.ricci@uniroma1.it; 3 Department of Pharmaceutical Medicine, Local Health Unit ‘Sud-Est’, ‘Misericordia’ Hospital, 58100 Grosseto, Italy; leonardogianluca.lacerenza@uslsudest.toscana.it (L.G.L.); elisa.petrucci@uslsudest.toscana.it (E.P.); 4 Cardiology Department, Local Health Unit ‘Sud-Est’, ‘Misericordia’ Hospital, 58100 Grosseto, Italy; alberto.cresti@uslsudest.toscana.it (A.C.); pasquale.baratta@uslsudest.toscana.it (P.B.); 5 Medical Management Department, Local Health Unit ‘Sud-Est’, ‘Misericordia’ Hospital, 58100 Grosseto, Italy; andrea.serafini@uslsudest.toscana.it; 6 Institute of Clinical Physiology, Italian National Research Council, 56124 Pisa, Italy; scalese@ifc.cnr.it

**Keywords:** triple therapy, single inhaler, multiple inhalers, COPD, exacerbations, oral corticosteroids, antibiotics, short-acting beta2 agonists

## Abstract

**Background:** Today, single-inhaler ICS/LAMA/LABA (SITT) COPD therapies are available. It is unclear whether they are more effective than multiple-device triple therapies (MITT) in improving COPD outcomes. **Methods**: We retrospectively considered patients on SITT/MITT in 2019/2020 who were prescribed >7 packages of ICS/LABA/LAMA or ICS/LAMA (+LAMA). The two treatments were compared concerning systemic corticosteroids, antibiotics, salbutamol, antifungal prescriptions, and number of emergency room visits/hospitalizations (ERV/Hs). We studied 292 MITT patients (Group A) during 2019, switching to SITT in 2020, and 366 subjects (Group B) who took SITT during 2019, and 206 MITT individuals (Group C) in 2020. **Results:** ICS/LABA + LAMA (MITT) package use was 8.2 ± 4.2 and 7.85 ± 4 in 2019, switching to 11.2 ± 3.2 when patients shifted to SITT in 2020 (*p* = 0.0001). Group A MITT package use was lower than in SITT patients in 2019 (9.31 ± 2.71, *p* = 0.0001; Group B). Throughout 2020, Group C (10.3 ± 6.1 packs) adherence to ICS/LABA was similar to SITT adherence in Group A (*p* = 0.270), whereas LAMA package use (9.1 ± 5) was lower (*p* = 0.0038). Patients using systemic corticosteroids/antibiotics were fewer in SITT in 2020, compared to the MITT results in 2019. Subjects with fewer ERV/Hs were observed with SITT rather than with MITT. Particularly, 13.8% of patients needed ≥2 ERV/Hs when treated with SITT, whereas 19.2% needed ≥2 ERV/Hs with MITT in 2019 (*p* = 0.08). Multivariate analysis, adjusted for all confounding factors including treatment adherence, showed that MITT (vs. SITT) can have an increased risk of at least one ERV/H (subjects receiving MITT during 2019 passing to SITT in 2020, OR: 1.718 [1.010–2.924], *p* = 0.046; Group A/MITT/2019 vs. Group B/SITT/2019, OR: 1.650 [0.973–3.153], *p* = 0.056; Group C/MITT/2020 vs. Group B/SITT/2019, OR: 1.908 [1.018–3.577], *p* = 0.044). **Conclusions:** SITT therapy may promote treatment adherence more effectively, therefore, reducing COPD exacerbations better than MITT. A possible synergistic effect of ICS/LABA/LAMA intake with a single device might be another cause of SITT’s greater efficacy.

## 1. Introduction

Patients with chronic obstructive pulmonary disease (COPD) who experience frequent exacerbations and/or more symptoms, despite being prescribed dual long-acting muscarinic antagonist (LAMA)/long-acting β2-agonist (LABA) or inhaled corticosteroid (ICS)/LABA therapies, are recommended triple ICS/LAMA/LABA treatment [1].

In the last few years, combinations containing ICS, LABA, and LAMA have been developed into a single device; these combinations offer, among other things, potential advantages in practicality and treatment adherence.

Triple fixed therapy in a single inhaler with ICS/LAMA/LABA has been demonstrated to be effective in improving lung function, symptoms, and health status, and in decreasing moderate/severe COPD exacerbations compared with ICS, LABA, or LAMA monotherapies and LAMA/LABA and ICS/LABA combinations [1,2,3,4,5,6]. Particularly, a meta-analysis with data obtained from 21,909 COPD patients, extracted from the ETHOS, KRONOS, IMPACT, and TRILOGY studies, has recently highlighted the superiority of ICS/LABA/LAMA in comparison to LABA/LAMA and ICS/LABA regardless of blood eosinophil count [3].

However, many patients are still being treated with triple therapy, using multiple devices several times a day with possible repercussions in terms of adherence and efficacy. To date, it is not clear whether triple therapy in a single inhaler is actually more effective than triple therapy with multiple devices.

Some observations indicate that triple therapy with a single inhaler (SITT) seems to give better results than triple therapy with multiple devices (MITT), consequently also leading to savings in terms of healthcare costs [7,8,9,10,11,12]. Conversely, another trial showed that there was no difference in effectiveness between a fixed and an open triple therapy [13].

Therefore, given the remaining uncertainties on this matter, we wanted to evaluate whether the adherence and efficacy obtained with SITT and MITT treatments could be different in COPD patients.

## 2. Materials and Methods

### 2.1. Study Subjects

From the pharmaceutical prescriptions archive database (used by South-Eastern Tuscany Central Pharmacy), we retrospectively extracted the patients who had received triple therapy in a single dispenser (SITT) with fluticasone furoate/vilanterol/umeclidinium (FF/UMEC/VI) (DPI—Dry-Powder Inhaler) or beclometasone dipropionate/formoterol fumarate/glycopyrronium (BDP/FF/GLI) (MDI—metered-dose inhaler) in 2019 and 2020. Among them, we only considered those who had been prescribed more than 7 triple therapy packages per year. We also examined patients performing triple therapy with multiple devices (MITT), especially those using ICS/LABA plus LAMA with different dispensers in the same period (2019–2020). We examined only subjects who had more than 7 ICS/LABA prescriptions/year, regardless of the number of associated LAMA packages. It should be noted that the packages of inhalers dispensed directly to patients in the pharmacy were considered. Therefore, direct collection of the drug would confirm the regularity of treatment. To exclude asthmatics treated with MITT and in order to make the MITT and SITT groups homogenous in terms of severity levels, we studied only patients who had received one SITT prescription in the year following the study period (identified by using tax codes). Prescribing SITT allowed us to identify subjects affected by COPD. In Italy, a pulmonary specialist can and must actually prescribe SITT with a therapeutic plan after having correctly performed a COPD diagnosis. Therefore, our database selection of patients, who had at least one SITT prescription, allowed us to identify those with a definite COPD diagnosis. However, all subjects selected in 2019/20 were already being treated with at least one of the inhaled drugs used in COPD treatment. Therefore, their disease had already been diagnosed before the study. Despite the 2020 lockdown, drug prescriptions were guaranteed online to all patients during the closure periods by family doctors.

The data analysis regarded 2019 and 2020, considering the two years separately, dividing patients by year of follow-up (as 2020 was the year in which the SARS-CoV-2 pandemic spread) in order to eliminate the possible influence of the COVID-19 period on the results.

The study was approved by the “Area Vasta Sudest Ethical Committee (C.E.A.S.V.E.), Azienda Ospedaliera Universitaria Senese and Azienda USL Toscana Sud-Est” (Protocol TRIPLECOPD, ID: 19196; determination: N° 358, 16 February 2021), on the basis that it complied with the declaration of Helsinki and that the protocol followed existing good clinical practice guidelines.

### 2.2. Study Design

The dispensing of drugs from chemists to patients, like systemic corticosteroids (ATC code: H02), antibiotics (ATC code: J01), salbutamol (SABA; ATC code: R03AC02), and antifungal medications, for each year considered, was also retrospectively taken into account for all patients by using tax codes to identify the subjects. Furthermore, hospitalizations and visits to emergency rooms due to COPD exacerbations during SITT and MITT for each year were similarly considered. Such data were also retrospectively retrieved from hospital discharge form databases by using the patients’ tax codes. All outcomes (systemic corticosteroids, antibiotics, salbutamol, antifungal medications, hospitalizations, and visits to emergency rooms) observed in COPD patients during one year of MITT and SITT were compared.

### 2.3. Methods

The systemic corticosteroids considered in this study, which can be used in COPD exacerbations, were Betamethasone, Dexamethasone, Methylprednisolone, Prednisolone, Prednisone, and Deflazacort. Antibiotics—like penicillins with extended action spectrums, combinations of penicillins with beta-lactamase inhibitors, cephalosporins, macrolides, fluoroquinolones, combinations of sulphonamides and trimethoprim, and some aminoglycosides—were pondered for this research, as they can also be used in COPD exacerbations. The antifungal medications studied were fluconazole, nystatin, miconazole, and itraconazole, used also for treating oral candidiasis.

ICD-9 codes of 490, 491, 492, 494, and 496 reported in hospital discharge forms, or 518.81–518.84, 786.0, 786.2, and 786.4, but associated with at least one of the secondary diagnoses with the codes 490–491–492–494–496, were regarded as hospitalizations for COPD exacerbations. Visits to emergency rooms were taken into account when the ICD-9 codes 490, 491, 492, 494, 496, 518.81–518.84, 786.0, 786.2, 786.4, 465.9, 466.0, 466.19, 487.1, 487.8, 493.90, and 493.91 were reported as in hospital discharge forms.

The ICS/LABAs considered were Fluticasone propionate/salmeterol, Fluticasone furoate/vilanterol, Budesonide/formoterol, and Beclomethasone/formoterol, while LAMAs were Tiotropium, Glycopyrronium, Umeclidinium, and Aclidinium.

The dispensing of maintenance inhaler medications >7 packages from chemists to patients was the cut-off that identified proper adherence to treatment. Individuals taking less than 7 ICS/LABA/LAMA or ICS/LABA packs per year were excluded from the study because they were either poorly adherent or because they had their therapy changed by switching to other inhaler therapies during the year of treatment.

Prescriptions of other drugs indicated for other diseases were also considered for each patient in order to trace the comorbidities that the individuals examined in this study might be affected by.

Patients’ lung function and some other clinical data were unknown as they came from different parts of the southeastern Tuscany area; therefore, it was impossible to find such data because they had been archived in various local databases that were not easily accessible. However, for a triple therapy prescription, a diagnosis of symptomatic moderate-to-severe COPD with a history of frequent and/or severe exacerbations (according to the GOLD guidelines) [1] was required. Such a prescription had to be made exclusively by a pulmonologist (not by a GP) with a written treatment plan as indicated by the Italian Drug Agency (AIFA). In addition, the COPD diagnosis had to be carried out with a spirometry.

### 2.4. Statistical Analysis

Paired *t*-tests were used to compare the number of packages of SITT and MITT taken by the same subjects in 2019 and 2020. The Mann–Whitney test was also used when the groups were different. Comparisons of the number of patients that required systemic corticosteroids, antibiotics, and antifungal medications in the two groups were performed by using the chi-square test. The same test was used to compare emergency room visits/hospitalizations observed during SITT and MITT treatments.

Since the patients of the different groups showed different adherence to SITT/MITT, a multivariate analysis was also performed. Logistic binary or multinomial regression models were applied to test whether a therapeutic regimen with MITT could determine a different risk of having at least one prescription of systemic corticosteroids, antibiotics, and SABA, as well as antifungal medications, when compared to SITT treatments. Logistic binary or multinomial regression models were also considered to test whether a therapeutic regimen with MITT could cause a different risk of having emergency room visits/hospitalizations (1 or ≥2) when compared to SITT treatments.

Models were all adjusted for sex, age, adherence (numbers of MITT or SITT packages, because they were different in the various groups), and comorbidities.

## 3. Results

**Figure 1:**
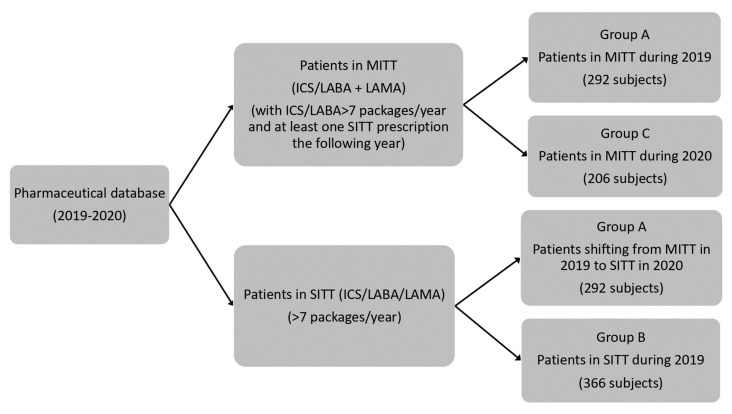
Study protocol. Patients treated with triple therapy with multiple devices (MITT) and with triple therapy with single devices (SITT). Inhaled corticosteroids (ICS), long-acting β2 agonist (LABA) and long-acting muscarinic antagonist (LAMA). SITT: ICS/LABA/LAMA (single inhaler); MITT: ICS/LABA + LAMA (triple therapy consisting of two devices, one for ICS/LABA and the other for LAMA).

The number of subjects who took MITT during 2019 and who switched to SITT in 2020 was 292 (Group A). Group B was characterized by patients (366) who took SITT during 2019. Individuals taking MITT during 2020 numbered 206 (Group C). Table 1 shows the reported characteristics of groups A, B, and C. No differences in number, sex, age, and comorbidities among groups were found (see study protocol in Figure 1).

### 3.1. Adherence

**Figure 2:**
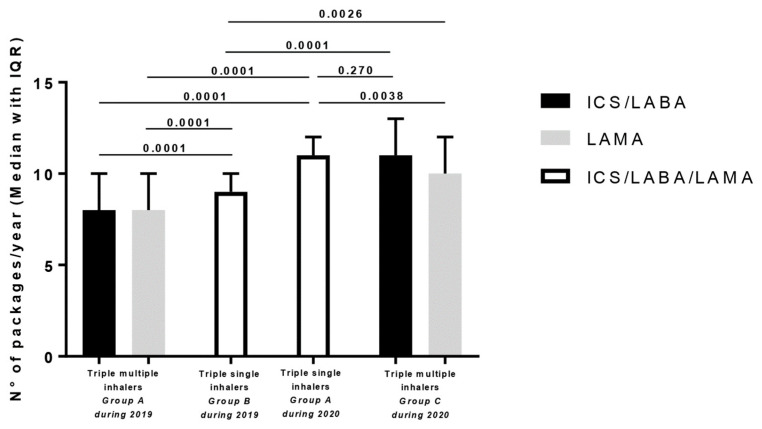
Adherence (number of packages/year) observed in the different groups treated with triple therapy with multiple devices (MITT) and with triple therapy with single devices (SITT) in 2019 or 2020.

When we considered Group A, the adherence to ICS/LABA and LAMA was at 8.2 ± 4.2 and 7.85 ± 4 packages during 2019, passing to 11.2 ± 3.2 boxes in the same subjects when they shifted to SITT (ICS/LABA/LAMA) in 2020, (*p* = 0.0001). The adherence observed during 2019 in subjects treated with MITT (Group A) was also lower in comparison with patients treated with SITT in 2019 (9.31 ± 2.71 packages, *p* = 0.0001; Group B). During 2020 in Group C, ICS/LABA adherence (10.3 ± 6.1 packs) was similar to that observed in the same year with SITT in Group A (*p* = 0.270), whereas the number of LAMA packages used (9.1 ± 5) was lower (*p* = 0.0038) in comparison. See Figure 2 for the graphical presentation of these results.

### 3.2. Use of Systemic Corticosteroids, Antibiotics, SABA, and Antifungal Medications during SITT and MITT

**Figure 3:**
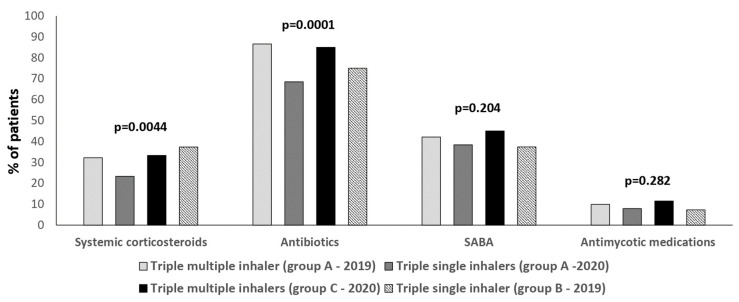
Percentages of subjects who had had at least one prescription of systemic corticosteroids, antibiotics, SABA (short-acting β2-agonist), and antifungal packages during 2019 and 2020 in the various groups considered.

The number of patients that received at least one prescription of systemic corticosteroids and antibiotics was lower in SITT subjects in 2020 (Group A; 68–23.3% and 200–68.5%) compared with MITT individuals in 2019 (Group A; 94–32.2% and 253–86.6%) and 2020 (Group C; 122–33.3% and 311–85%; *p* = 0.0044 and *p* = 0.0001; Figure 3). No differences in the use of SABA and antifungal medications in both SITT and MITT patients were found (Figure 3).

### 3.3. Emergency Room Admissions and Hospitalizations

**Figure 4:**
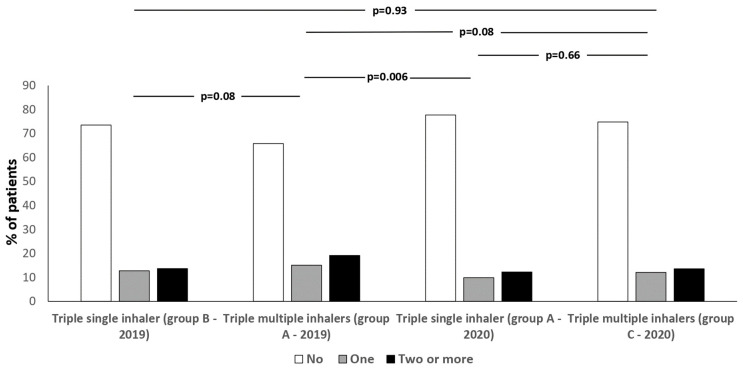
Percentages of subjects who had one or at least two emergency department visits/hospitalizations during one year of MITT or SITT therapy (2019 or 2020) in the various groups considered.

When the same subjects who took MITT in 2019 switched to SITT in 2020, the number of individuals with emergency room visits/hospitalizations due to COPD exacerbations was significantly reduced. In fact, 44 (15.1%) and 56 (19.2%) patients had at least 1 or >2 emergency department visits/hospitalizations during 2019, while the number dropped to 29 (9.9%) and 36 (12.3%) in 2020 (*p* = 0.006; Figure 4). Even when individuals who took SITT in 2019 were compared with those who took MITT in the same year (the pre-COVID-19 period), the number of subjects who accessed emergency rooms or were hospitalized was lower with the first treatment (*p* = 0.08; Figure 4). Likewise, the number of patients requiring emergency department visits/hospitalizations was higher in Group A with MITT during 2019 when compared to Group C with MITT during 2020 (*p* = 0.08; Figure 4). The number of subjects requiring emergency department visits/hospitalizations was similar in Group B in SITT during 2019 and Group C in MITT in 2020 (*p* = 0.93; Figure 4).

### 3.4. Multivariate Analysis on COPD Exacerbation Risk

**Figure 5:**
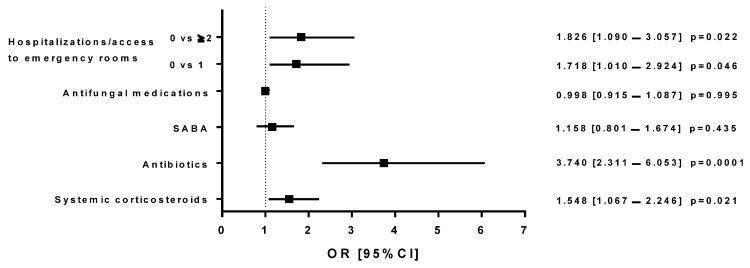
Logistic regression models that compared the effects (on different outcomes) of the treatment with triple therapy with multiple inhalers performed in 2019 and triple therapy with single inhalers prescribed in 2020 in the same subjects (Group A); The figure shows the odds ratios with a 95% confidence interval (OR [95% CI]).

**Figure 6:**
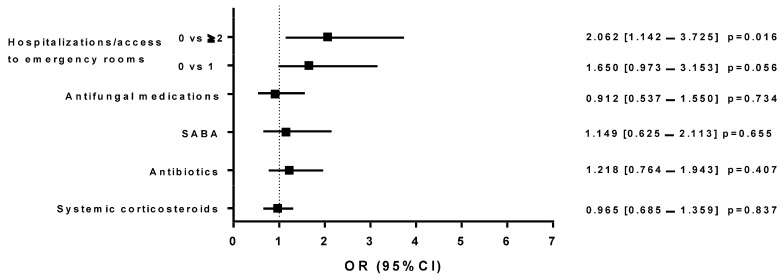
Logistic regression models that compared the effects (on different outcomes) of the treatment with triple therapy with multiple inhalers prescribed in Group A in 2019 and triple therapy with single inhalers in Group B in 2019; The figure shows the odds ratios with a 95% confidence interval (OR [95% CI]).

**Figure 7:**
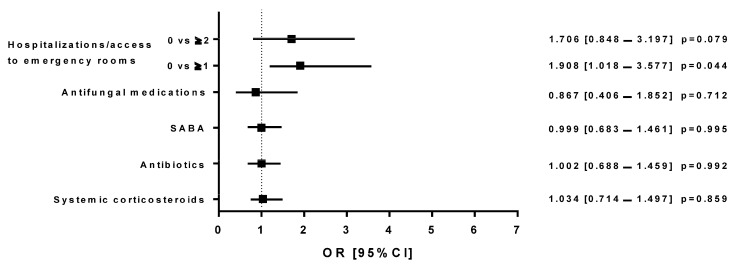
Logistic regression models that compared the effects (on different outcomes) of the treatment with triple therapy with single inhalers prescribed in Group B in 2019 and triple therapy with multiple inhalers in Group C in 2020; The figure shows the odds ratios with a 95% confidence interval (OR [95% CI]).

Group A patients receiving MITT in 2019, when compared with the same subjects who switched to SITT during 2020, had an increased risk of using systemic corticosteroids (OR: 1.510 [1.007–2.265]; *p* = 0.046) or antibiotics (OR: 3.740 [2.311–6.053]; *p* = 0.0001), and a greater risk of needing emergency room visits/hospitalizations (0 vs. at least 1, OR: 1.718 [1.010–2.924]; *p* = 0.046 and 0 vs. ≥2, OR: 1.826 [1.090–3.057]; *p* = 0.022; Figure 5). When MITT (Group A) and SITT (Group B) individuals were compared in 2019, an increased risk of emergency room visits/hospitalizations was observed with MITT (0 vs. at least 1, OR: 1.650 [0.973–3.153]; *p* = 0.056 and 0 vs. ≥2, OR: 2.062 [1.142–3.725]; *p* = 0.016; Figure 6). The same was highlighted in MITT subjects in 2020 (Group C), when compared to SITT patients (Group B) in 2019, who showed an increased risk of needing emergency room visits/hospitalizations (0 vs. at least 1, OR: 1.908 [1.018–3.577]; *p* = 0.044; Figure 7).

## 4. Discussion

This study highlighted that a one-year single-inhaler triple therapy seems to be more effective than a multiple-inhaler triple treatment in reducing the use of systemic corticosteroids/antibiotics (exacerbation surrogates), and, especially, emergency room visits/hospitalizations for COPD exacerbations. The data analysis covered 2019 and 2020, considering the two years separately—as 2020 was the year in which the SARS-CoV-2 pandemic spread—with the aim of eliminating a possible influence of the COVID-19 period on the results. In fact, several studies have suggested that the 2019 SARS-CoV-2 pandemic was associated with a decreased rate of acute exacerbations of chronic obstructive pulmonary disease (AECOPD) in 2020. Lockdown restrictions associated with universal masking and social distancing, a reduction in air pollution during this period, and the improvement in adherence of inhaled treatment appear to be connected with a reduction in severe COPD exacerbations [14,15,16,17,18,19]. Furthermore, medical activities in favor of outpatients were suspended for some months in 2020 due to the pandemic. Therefore, milder forms of AECOPDs were probably self-managed by patients and were not presented to the healthcare system during the lockdown.

Subjects who switched from MITT (prescribed in 2019) to SITT in 2020 showed a significant reduction in the use of corticosteroids/antibiotics, as well as emergency room visits/hospitalizations for COPD exacerbations, confirming greater SITT effectiveness. However, as already stated, 2020 was characterized by the SARS-CoV-2 pandemic, which could have affected the results. The above-mentioned lockdown with social distancing, mask wearing, increased adherence to inhalers, and greater use of home, rather than hospital, care resulted in a reduction in COPD exacerbations/hospitalizations [14,15,16,17,18,19]. Therefore, the COVID-19 period, rather than favoring the greater efficacy of triple therapy in a single inhaler, might have been responsible for the reduction in disease exacerbations. Indeed, SITT adherence in 2020 increased significantly (by approximately 30%) compared to what was achieved with MITT in 2019, probably due also to the anxiety induced by the pandemic. Therefore, it was not only lockdown restrictions, the use of a mask, and/or improved COPD self-management, but also this better adherence, that may have improved treatment responses attained with SITT and the various outcomes considered by our study. Indeed, many researchers have confirmed that a higher adherence improved all COPD outcomes [20,21,22]. Conversely, our study also observed that the group of subjects who took SITT during 2019 (before the pandemic) showed a higher adherence than those who took MITT in the same year with a consequent reduction in exacerbations/hospitalizations. This confirms that single-inhaler therapy favors treatment adherence in contrast with the use of multiple inhalers with a consequent greater effectiveness, regardless of the possible SARS-CoV-2 influence. Other studies have confirmed that the use of the triple therapy with a single inhaler, rather than with multiple devices, improved adherence [22,23,24], which can lead to higher effectiveness in COPD outcomes, thus confirming that regular/continuous treatment is one of the main factors influencing therapeutic efficacy, as highlighted by other studies [25,26,27]. This is also shown by the improvement in the results obtained with MITT in 2020 (Group C) where treatment adherence was greater than the one observed with SITT and MITT in 2019. Therefore, one of the objectives that must be set for inhalation therapy is to ensure regular adherence to the therapy, which seems to be more easily achieved with single-inhaler triple therapy. Furthermore, although a better MITT adherence was observed in 2020 (because of the anxiety induced by COVID-19), it appeared to be higher for ICS/LABA but lower for LAMAs. This suggests that when two inhalers are prescribed, LAMA might be less used than ICS/LABA, which could be due to the slowness of bronchodilation induced by LAMAs, compared to LABAs, thus leading to a possible reduced intake of anticholinergics caused by a lower perception of efficacy with the latter medications. This may contribute to a lower effectiveness for MITT.

When the multivariate analysis was applied, thus eliminating the possible influence of treatment adherence, we observed that the risk of access to emergency rooms rooms/hospitalizations, which was also corrected for MITT/SITT packages, was higher in MITT subjects, regardless of whether the period analyzed was before or during the COVID-19 pandemic. This would lead us to hypothesize that SITT may have greater efficacy in terms of a reduction in exacerbations regardless of treatment adherence. There might be an additional mechanism enhancing the efficacy of triple therapies in a single inhaler. A synergistic effect due to the simultaneous administration of drugs may be one of the mechanisms underlying the greater effectiveness of a single-inhaler therapy compared to the one obtained via multiple devices. We know that bronchodilator combinations (LAMAs and LABAs) have also been demonstrated to exhibit a superior efficacy due to their synergistic action mode when compared to monotherapy [28,29,30]. This has also been demonstrated with the triple beclomethasone/formoterol/glycopyrronium combination that induced a synergistic bronchorelaxant effect in medium and small human airways [31]. Consequently, the simultaneous intake of inhaler medications should always be considered in COPD treatment.

It should also be added that the higher adherence (plus the synergy of the three drugs dispensed by the same device) might have positive repercussions, not only on clinical efficacy, but also in terms of lower healthcare costs [10,11,32,33]. This should always be taken into account when prescribing triple therapy, always giving priority to single-inhaler treatment.

In contrast to what is highlighted by our study, a trial observed that the results obtained with a single-inhaler triple therapy were similar to an open triple therapy (ICS/LABA + LAMA), both in terms of the increase in FEV1 and above all in the reduction in moderate-to-severe exacerbations [13]. According to our research, a real-life setting—a very heterogeneous population characterized by various comorbidities and a different adherence to treatments—may have led to better outcomes with single-inhaler triple therapy. In fact, as we have already stated, adherence to LAMA treatment in real life could be lower when such medication is used with a different/separate device from the one containing also ICS/LABA, thus reducing effectiveness of multiple-inhaler triple therapy.

A limitation of this study is the lack of lung function data, which prevents the categorization of patients according to disease severity. However, following Italian legislation, triple therapy in a single device must be prescribed by a pulmonologist to subjects with a moderate/severe COPD diagnosis (obtained also via spirometry) as required by the GOLD guidelines. Consequently, the selection of such patients makes it unlikely for the disease severity to be different in the various patient groups. Furthermore, individuals who were chosen in the MITT group were those who had received a SITT prescription during the year after the study period. This allowed us to select MITT patients who had similar COPD severity levels compared to those in the SITT group. Thus, this type of selection would make the groups homogeneous in terms of lung function.

## 5. Conclusions

To sum up, regardless of the possible influence of COVID-19, single-inhaler triple therapy seemed to be more effective than multiple-inhaler treatment, especially in reducing COPD exacerbations. The increased adherence induced by SITT could be one of the main reasons for this increased efficacy. A further reason might be the simultaneous intake of the drugs contained in a single inhaler, which could induce a synergistic bronchodilator effect with possible repercussions on therapeutic effectiveness. This study appears to indicate that it would be advisable to lean towards a choice of a single-inhaler triple therapy rather than the use of multiple devices for the treatment of COPD patients.

## Figures and Tables

**Table 1:** Characteristics of COPD patients treated with triple therapy with multiple devices (MITT) and/or with triple therapy with single devices (SITT) in 2019 or 2020. Group A (292 patients) treated with MITT and SITT in 2019 and 2020, respectively; Group B (366 patients) with SITT in 2019; Group C (206 patients) with MITT in 2020. No differences were found among the different groups for any of the parameters considered (*p* > 0.05).

	Group A	Group B	Group C
Sex (M/F)	191/101 (65.4/34.6%)	239/127 (65.3/34.7%)	124/82 (60.2/39.8%)
Age (mean ± SD)	75.5 ± 8.4	74.7 ± 8.9	74 ± 9.25
Gastropathy	198 (67.8%)	244 (66.7%)	127 (62%)
Bowel disease	33 (11.3%)	32 (8.7%)	17 (8.3%)
Diabetes	57 (19.5%)	76 (20.8%)	37 (18%)
Osteoporosis	26 (8.6%)	28 (7.7%)	17 (8.3%)
Heart and vascular diseases	221 (75.7%)	259 (70.8%)	147 (71.4%)
Anemia	43 (14.7%)	38 (10.4%)	19 (9.2%)
Renal failure	9 (3.1%)	8 (2.2%)	2 (1%)
Hypertension	205 (70.2%)	245 (67%)	137 (66.5%)
Dyslipidemia	96 (32.9%)	92 (25.1%)	55 (26.7%)
Prostate diseases	70 (24%)	86 (23.5%)	50 (24.3%)
Dysthyroidism	26 (8.9%)	24 (6.6%)	25 (12.1%)
Cancer	62 (21.2%)	91 (24.9%)	45 (21.8%)
Autoimmune diseases	10 (3.4%)	14 (3.8%)	8 (3.9%)
Hyperuricemia	72 (24.7%)	79 (21.6%)	47 (22.8%)
Neurological diseases	46 (15.8%)	45 (12.3%)	28 (13.6%)
Psychiatric diseases	89 (30.5%)	116 (31.7%)	64 (31%)
Glaucoma	27 (9.2%)	34 (9.3%)	21 (10.2%)

## Data Availability

The data are not available due to ethical and regulatory restrictions.
